# Impact of an open lung approach on hemodynamic parameters after cardiac surgery

**DOI:** 10.1186/cc10730

**Published:** 2012-03-20

**Authors:** A Leme, F Galas, M Volpe, J Fukushima, J Almeida, R Ianotti, L Hajjar, M Amato

**Affiliations:** 1 Heart Institute, São Paulo, Brazil

## Introduction

Lung recruitment maneuver (RM) has been associated with an increase of arterial oxygen saturation and improvement of respiratory parameters. Nevertheless, adverse hemodynamic effects can occur due to the RM technique. The aim of this study is to evaluate the effect of the RM on hemodynamic parameters in the immediate postoperative period after cardiac surgery.

## Methods

A total of 120 patients with PaO_2_/FiO_2 _ratio <250 was randomized to a conventional strategy of mechanical ventilation or open lung strategy. The open lung strategy was performed using RM with an inspiratory pressure amplitude of 15 cmH_2_O and PEEP of 30 cmH_2_O three times during 1 minute and setting PEEP after RM at 13 cmH_2_O. The conventional strategy was done using PEEP = 8 cmH_2_O and RM with CPAP = 20 cmH_2_O three times during 30 seconds and setting PEEP after RM at 8 cmH_2_O. The heart rate, systolic, diastolic and mean arterial blood pressures were recorded before, immediately and 5 minutes after RM. Respiratory mechanics and blood gas analysis were recorded before and after RM.

## Results

The open lung group presented a higher variability on blood pressure immediately after RM compared to the conventional group. There were no differences in baseline blood pressure or 5 minutes after RM and heart rate between groups. The open lung group presented higher lung compliance (60 ± 17 vs. 48 ± 13 ml/cmH_2_O) and PaO_2_/FiO_2 _(431 ± 124 vs. 229 ± 68) ratio compared to the conventional group.

## Conclusion

An open lung approach after cardiac surgery improves lung compliance and the PaO_2_/FiO_2 _ratio with minimum hemodynamic detrimental effect.
